# Large Cutaneous Squamous Cell Carcinoma: A Consequence of Elective Surgery Deferment During the COVID-19 Pandemic

**DOI:** 10.7759/cureus.48195

**Published:** 2023-11-03

**Authors:** Andrew Hess, Kanica Yashi, Jaswinder Virk, Amrat Kumar, FNU Meena

**Affiliations:** 1 Internal Medicine, Bassett Healthcare, Cooperstown, USA; 2 Cardiology, Bassett Healthcare, Cooperstown, USA

**Keywords:** cutaneous squamous cell cancer, face tumor, covid 19, ­skin cancer, squamous cell carcinoma (scc)

## Abstract

Cutaneous squamous cell carcinoma (cSCC) is a common skin cancer that can be treated effectively with limited morbidity if caught and treated early with elective surgical procedures. The COVID-19 pandemic caused most healthcare facilities to delay or defer elective surgeries as they allocated workforce and resources to treating significant increases of critically ill patients. This care delay has increased morbidity and mortality of many conditions treated with surgery. A few case reports exist on delayed elective surgeries' effects on patients and healthcare facilities. We report a case of cSCC enlarging and locally spreading due to elective surgery delay during the COVID-19 pandemic.

## Introduction

Cutaneous squamous cell carcinoma (cSCC) is a common dermatological cancer that most often occurs on sun-exposed skin in fair-skinned individuals. Clinically, it has a variety of appearances, including but not limited to crusted nodules, open sores with raised edges, scaly erythematous patches, and wartlike lesions. It is definitively diagnosed with a biopsy, and early surgical excision of cSCC lesions significantly reduces morbidity and mortality [1]. Many elective surgical procedures, including surgical excisions of skin lesions, were delayed and deferred while healthcare facilities became overwhelmed with critically ill patients during the COVID-19 pandemic. Patients were lost to follow-up owing to their apprehension about contracting COVID-19 during doctor visits or because their care providers were overwhelmed with a surge of critically ill patients, leading to prolonged postponements in their care. This delay in care caused physical and emotional harm to patients with various conditions treated with elective surgeries. We present a case of an extensive and invasive cSCC in a 65-year-old male who had treatment delayed during the COVID-19 pandemic [2].

## Case presentation

**Figure 1:**
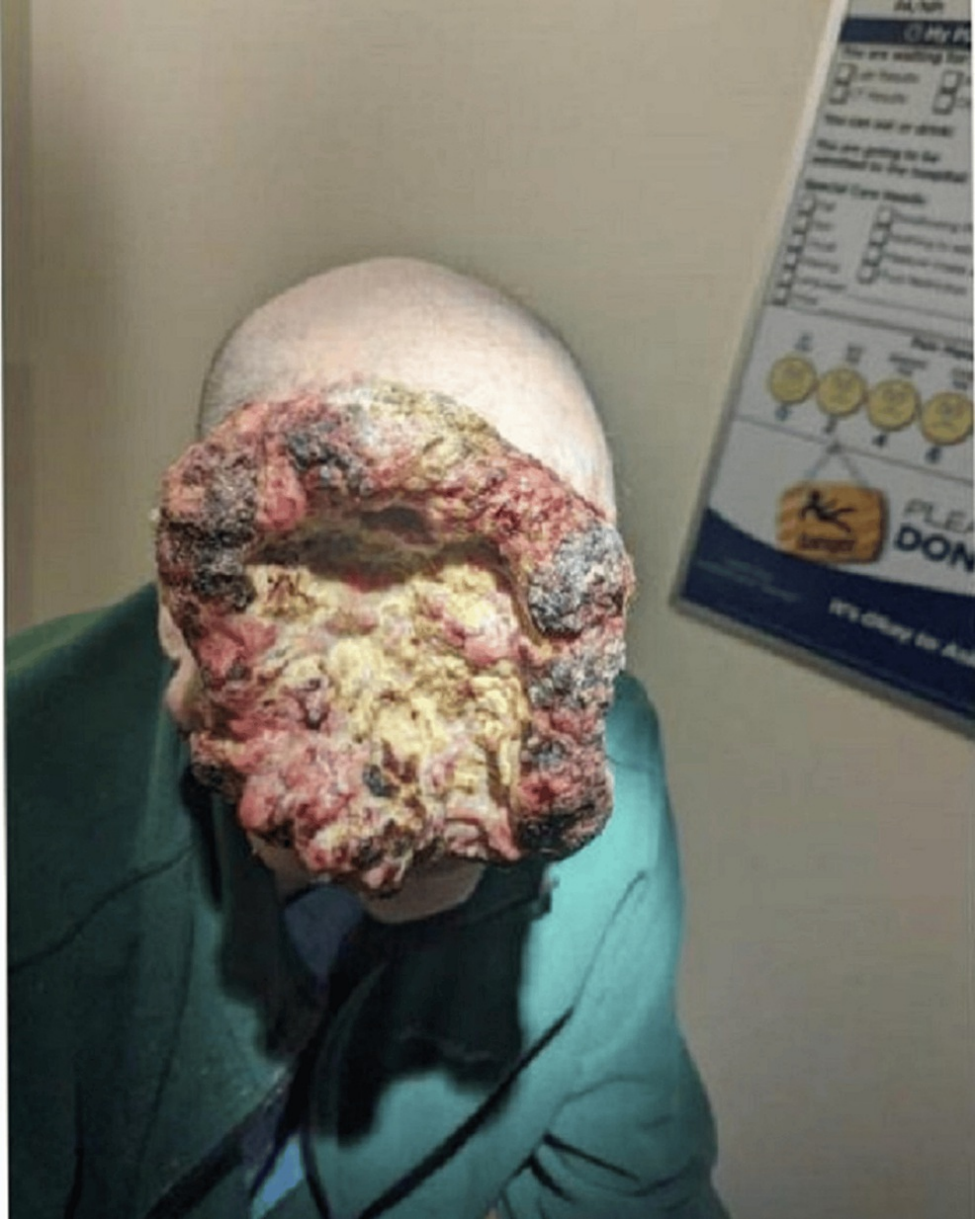
Large mass with raised "cauliflower-like" edges, darkened scabs likely from capillary bleeds, and a depressed, yellow necrotic center

**Figure 2:**
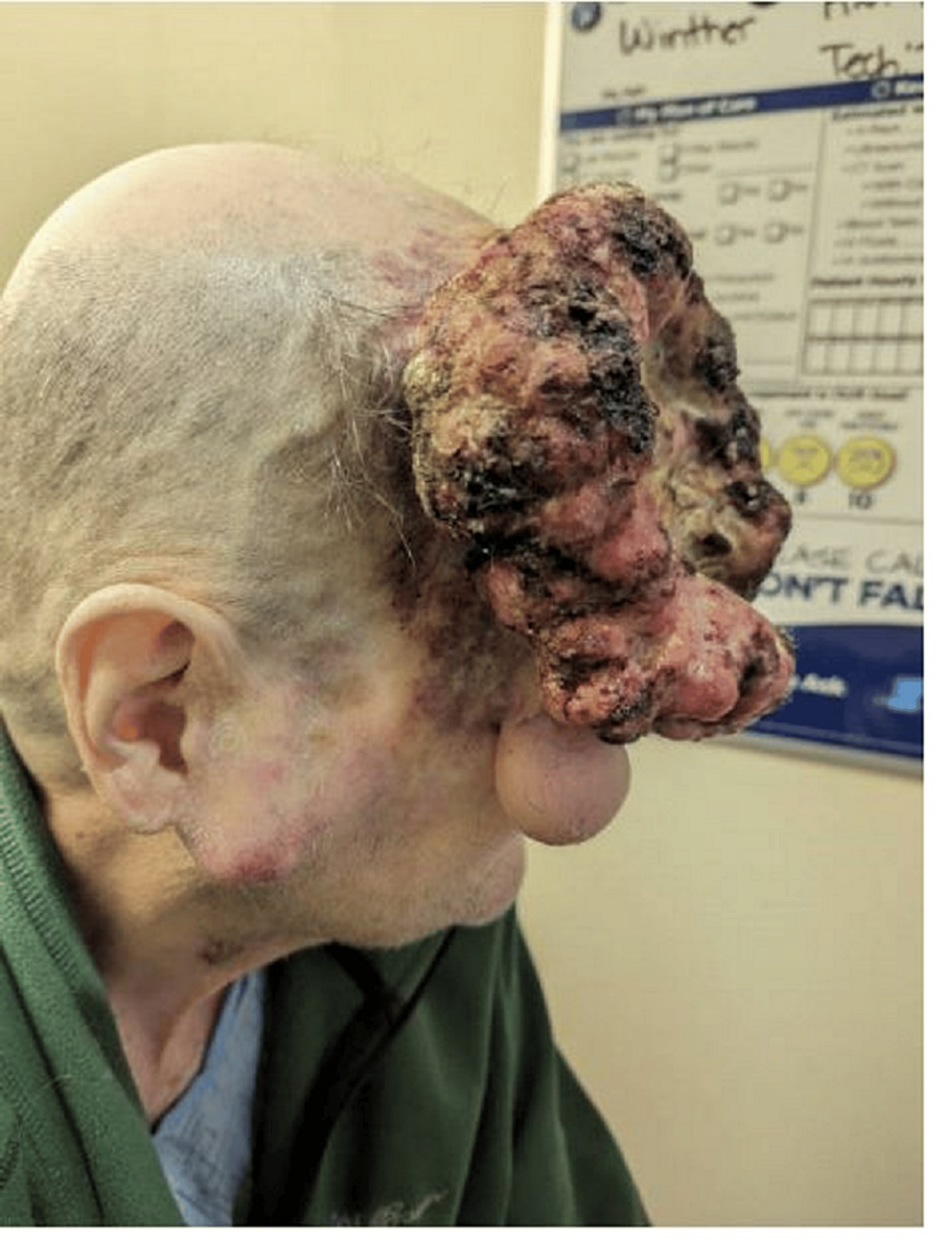
Lateral view visualizes large main mass, edematous right lower eyelid, hard right parotid mass, and flat dry lesion

A 65-year-old male with a past medical history of squamous cell carcinoma on his scalp status post excision in 1990 presented to our hospital with significant anemia. He was found to have a large right-sided facial mass and another smaller mass over his right parotid gland. He first noticed a small, nickel-sized, indurated mass on his forehead in 2020. The mass slowly grew, for which he tried to seek medical care several times. During the COVID-19 pandemic, hospitals were deferring elective surgeries; thus, timely care could not be provided to him in any hospital. At this point, he became frustrated and attempted to remove the mass himself at home with a knife. On the first attempt, he saw no active bleeding and noticed the wound had healed. However, the mass grew back and more rapidly this time. He attempted to cut it off again, but it started to bleed significantly; therefore, the patient did not self-intervene further. The mass continued to grow over the next few weeks involving the right parotid gland and neck. Around this time, the patient sustained a fall at home and hit the mass, causing it to bleed. He visited his primary care physician feeling tired and weak. His blood work revealed a hemoglobin of 5.5 and was sent to the emergency department. On presentation, his vitals showed: blood pressure of 124/77 mmHg, pulse of 107 beats per minute, respiration rate of 16 breaths per minute, oxygen saturation of 100% on room air, and temperature of 98.6 F. Lab work was significant for hemoglobin of 5.8 and a hematocrit of 19.9. On physical exam, he was alert and oriented, with a large mass on the right side of his face and scalp. Its dimensions were approximately 20 cm horizontally and 16 cm vertically. It had cauliflower-like raised edges with a large depressed central necrotic area (Figures 1-2).

**Figure 3:**
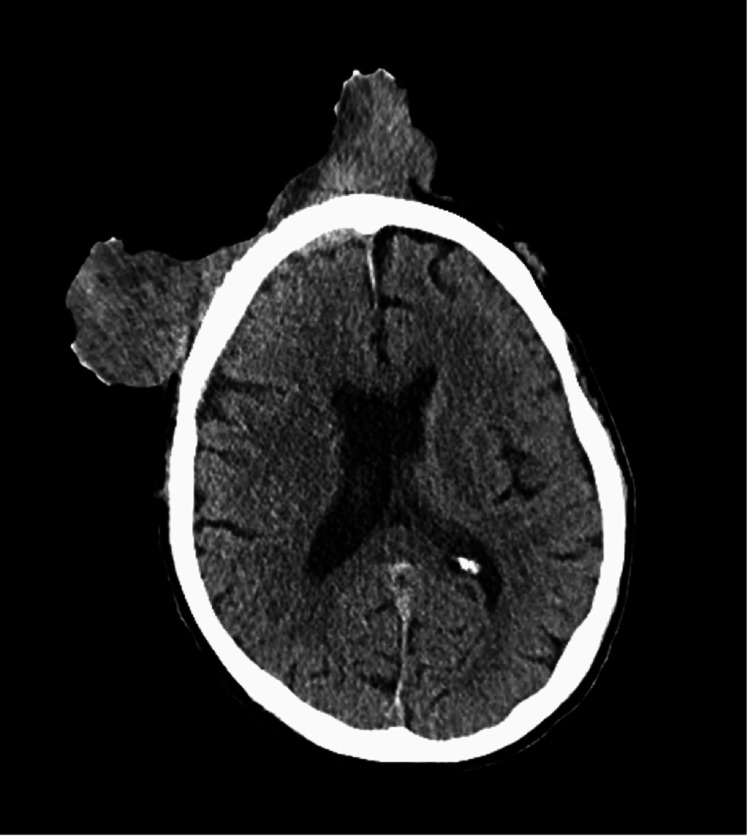
CT head with contrast showing an enhancing penetrating mass in the right frontal extra-axial space

**Figure 4:**
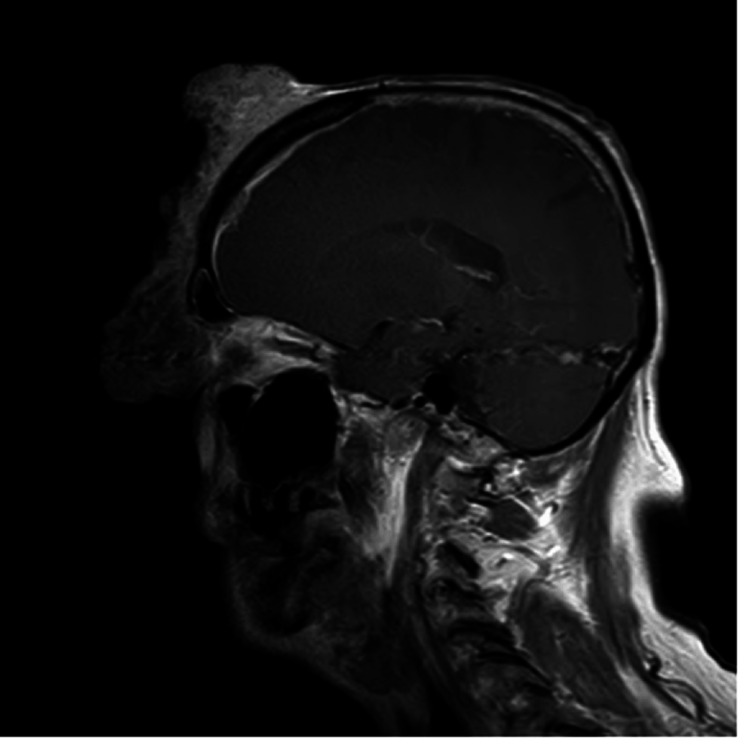
MRI face T1-weighted post contrast sagittal view of enhancing mass in the right frontal extra-axial space

It was malodorous with multiple areas of chronic capillary bleeding. The mass completely obstructed his right eye and caused severe edema of his eyelids. His vision was found to be fully intact in his right eye, but the mass was obstructing his vision. A firm mass was found over his right parotid gland. The rest of his physical exam was normal. Multiple imaging studies were done of the mass, including an MRI of the face and brain and a CT of the head and neck. This revealed a large malignant right frontal/facial mass that penetrated the calvarium into the right frontal extra-axial space and right frontal sinus. CT neck and MRI face showed a 3.4 x 4.0 x 4.3 cm mass over the right parotid gland (Figures 3-4).

His severe anemia was treated by transfusing him with four units of packed red blood cells, bringing his hemoglobin back up to 9.0. The surgical team biopsied the large facial mass in multiple locations, including the margins and the parotid mass. Pathology from all biopsy sites came back consistent with squamous cell carcinoma. He was discharged after stabilizing his anemia. At this time, all the initial imaging of the mass was also complete. A follow-up CT chest, abdomen, and pelvis was performed as an outpatient and showed no metastatic disease. His case was discussed by a multidisciplinary tumor board, which concluded that the best course of action would be to remove as much of the facial and parotid mass as possible with surgery and then treat the affected areas with radiation and immunotherapy. Following this, a PET CT would be required to facilitate radiation therapy planning. It was felt that enucleation of the right eye would likely be required, and he would likely lose his right facial nerve, resulting in permanent right facial droop.

## Discussion

cSCC is a malignant tumor that arises from epidermal keratinocytes, most commonly in sun-exposed areas and individuals with lightly pigmented skin. The lesions can appear as "wart-like bumps," crusted nodules, or rough and scaly erythematous patches. Clinical findings can suggest cSCC, but a histopathologic examination is needed to confirm the diagnosis. The size, location, and histopathological findings can help determine if the cSCC is at low or high risk of locally spreading and/or metastasizing [1,3-5]. The National Comprehensive Cancer Network 2020 guidelines define high-risk cSCC based on the following clinical features [3]: lesions of any size located on the head, neck, hands, feet, pretibial, and anogenital areas, lesions >2 to 4 cm located on the trunk or extremities (excluding pretibial, hands, and feet), and recurrent tumors.

Lesions with these clinical features should be biopsied and treated promptly to avoid increased morbidity and mortality. Treatment begins with surgical removal of the lesion with Mohs micrographic surgery or standard excision with wide margins, ensuring the margins are clear on pathology. The surgical removal is followed by possible adjuvant radiation and/or chemotherapy for lesions with multiple risk factors. Delaying treatment likely results in the growth and spread of the cancer [1,3,5].

During the novel COVID-19 pandemic, most elective surgeries, such as Mohs surgeries to treat cSCCs, were delayed as hospitals and clinics allocated resources to treat and prevent the spread of COVID-19. Postponement of most elective surgeries caused the worsening of certain medical conditions which would otherwise be treated. During the pandemic, the ambiguity surrounding the timing of procedures, combined with the anxiety of potentially worsening health conditions, took a substantial emotional and physical toll on patients [2,4,6]. This was the case with our patient, whose care was delayed during the COVID-19 pandemic [7,8].

## Conclusions

cSCC is a common skin cancer that can be cured when recognized early. Elective surgery deferments during the COVID-19 pandemic caused delays in proper treatment for this case of cSCC, leading to cancer enlargement and invasion into local structures requiring more invasive surgeries that would likely result in permanent neurological damage to his face and eye. Better follow-up and communication from his healthcare providers could have allowed for quicker treatment of his cancer and a subsequent reduction in morbidity. Knowledge about this case can be instrumental in understanding the consequences of elective surgery delays and developing new policies that will facilitate primary care and other outpatient facilities to continue providing care to patients with non-emergent illnesses during the global health crisis.
